# 1-(2-Hydr­oxy-3,4-dimethoxy­phen­yl)-2-(4-methoxy­phen­yl)ethanone

**DOI:** 10.1107/S1600536808036258

**Published:** 2008-11-13

**Authors:** Zhu-Ping Xiao, He-Ying Xiao

**Affiliations:** a College of Chemistry & Chemical Engineering, Jishou University, Jishou 416000, People’s Republic of China

## Abstract

In the title compound, C_17_H_18_O_5_, the pyrogallol group is almost coplanar with the mean plane of the attached carbonyl group [dihedral angle of 1.95 (13)°] and makes a dihedral angle of 56.01 (10)° with the other benzene ring. Of the three meth­oxy groups, only one is significantly twisted relative to its attached benzene ring [C—O—C—C torsion angles of 4.0 (5), 3.9 (6) and −106.3 (4)°]. Intra­molecular O—H⋯O and C—H⋯O hydrogen bonds help to establish the conformation, and the packing is consolidated by C—H⋯O inter­actions and π–π stacking interactions [centroid–centroid separation = 3.735 (2) Å].

## Related literature

For background on the properties of deoxy­benzoins, see: Kiuchi *et al.* (1990 ▶); Li *et al.* (2008 ▶); Niwa *et al.* (1999 ▶); Papoutsi *et al.* (2007 ▶); Parmar *et al.* (1996 ▶); Sanduja *et al.* (1985 ▶); Xiao *et al.* (2008 ▶); Xiao, Fang *et al.* (2007 ▶); Xiao, Shi *et al.* (2007 ▶).
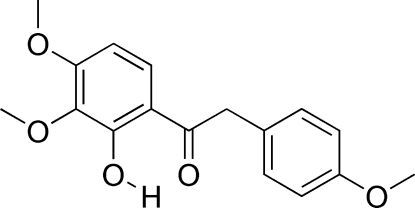


## Experimental

### 

#### Crystal data

C_17_H_18_O_5_
*M* *_r_* = 302.31
Monoclinic, 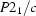

*a* = 14.431 (3) Å
*b* = 14.073 (3) Å
*c* = 7.4610 (15) Å
β = 92.86 (3)°
*V* = 1513.3 (5) Å^3^
*Z* = 4
Mo *K*α radiation
μ = 0.10 mm^−1^
*T* = 293 (2) K
0.30 × 0.30 × 0.30 mm

#### Data collection

Enraf–Nonius CAD-4 diffractometer
Absorption correction: ψ scan (North *et al.*, 1968 ▶) *T*_min_ = 0.971, *T*_max_ = 0.971
2893 measured reflections
2667 independent reflections
1543 reflections with *I* > 2σ(*I*)
*R*_int_ = 0.059

#### Refinement

*R*[*F* ^2^ > 2σ(*F* ^2^)] = 0.071
*wR*(*F* ^2^) = 0.215
*S* = 1.03
2667 reflections
206 parameters
H atoms treated by a mixture of independent and constrained refinement
Δρ_max_ = 0.28 e Å^−3^
Δρ_min_ = −0.35 e Å^−3^

### 

Data collection: *CAD-4 Software* (Enraf–Nonius, 1989 ▶); cell refinement: *CAD-4 Software*; data reduction: *XCAD4* (Harms & Wocadlo, 1995 ▶); program(s) used to solve structure: *SHELXS97* (Sheldrick, 2008 ▶); program(s) used to refine structure: *SHELXL97* (Sheldrick, 2008 ▶); molecular graphics: *SHELXTL* (Sheldrick, 2008 ▶); software used to prepare material for publication: *SHELXTL*.

## Supplementary Material

Crystal structure: contains datablocks global, I. DOI: 10.1107/S1600536808036258/hb2823sup1.cif

Structure factors: contains datablocks I. DOI: 10.1107/S1600536808036258/hb2823Isup2.hkl

Additional supplementary materials:  crystallographic information; 3D view; checkCIF report

## Figures and Tables

**Table 1:** Hydrogen-bond geometry (Å, °)

*D*—H⋯*A*	*D*—H	H⋯*A*	*D*⋯*A*	*D*—H⋯*A*
O2—H18⋯O1	0.89 (4)	1.78 (4)	2.583 (4)	148 (4)
C15—H15*A*⋯O2	0.96	2.56	3.086 (5)	115
C11—H11⋯O3^i^	0.93	2.51	3.259 (4)	138
C17—H17*B*⋯O4^ii^	0.96	2.59	3.315 (6)	133

Symmetry codes: (i) 
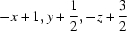
; (ii) 
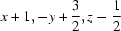
.

**Fig. 1.**
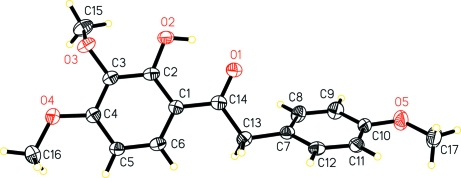
The molecular structure of (I) with displacement ellipsoids for the non-hydrogen atoms drawn at the 30% probability level.

## supplementary crystallographic information

### Comment

Deoxybenzoins are intermediates in the synthesis of isoflavones (Xiao *et al.*, 2008; Xiao, Fang *et al.*, 2007) and found in several plants, such as Glycyrrhiza *sp*., Trifolium subterraneum and Ononis spinosa, and marine sources (Kiuchi *et al.*, 1990; Niwa *et al.*, 1999; Sanduja *et al.*, 1985). Many of the deoxybenzoins have shown quite significant estrogen receptor modulatory, urease inhibitory, antimicrobial and antiviral properties (Papoutsi *et al.*, 2007; Xiao, Shi *et al.*, 2007; Parmar *et al.*, 1996; Li *et al.*, 2008). Consequently, we have synthesized a series of deoxybenzoins for bioactivity screen.

The bond lengths and angles of the title compound, (I), are unexceptional. The carbonyl group is almost coplanar with the pyrogallol fragment with a dihedral angle of 1.95 (13) °. The C16—O4—C4—C5 torsion angle [4.0 (5) °] indicates that the methoxy group is coplanar with the attached phenyl ring (pyrogallol fragment), the same as the methoxy group (O5, C17) attached to the *p*-methoxyphenyl moiety.  However, the C15—O3—C3—C4 torsion angle indicates that the methoxy group is twisted at -106.3 (4) ° with respect to the pyrogallol ring.

The molecule of (I) is stabilized by intramolecular O—H···O and C—H···O hydrogen bonds (Table 1). The six-membered pseudo-ring closed by the former bond is almost planar with mean deviation of 0.010Å and coplanar with the fused C1—C6 benzene ring (Fig. 1).  The packing is stabilized by intermolecular C—H···O hydrogen bonds as well as weak π-π interactions with a centroid-to-centroid distance of 3.735 (2)Å.

### Experimental

4-Methoxyphenacetyl chloride was prepared by treating 4-methoxyphenacetic acid with thionyl chloride at room temperature for 24 h. The solvent was removed *in vacuo*. To a mixture of pyrogallol trimethoxyl ether (2 g, 11.9 mmol) and 4-methoxyphenacetyl chloride (2.3 g, 12.2 mmol) in dried carbon disulfide (4 ml) was added anhydrous aluminium chloride (2.8 g) with stirring at room temperature overnight. Then the mixture was heated under reflux for 0.5 h. After removal of the solvent by decantation, the residue was poured into ice-cold diluted hydrochloric acid. The precipitate was suction filtered and crystallized from methanol to furnish light yellow blocks of (I).

### Refinement

The H atom bonded to O2 was located in a difference Fourier map and freely refined. All other H atoms were placed in geometrically idealized positions (C—H = 0.93-0.97Å) and refined as riding with *U*_iso_ = 1.2*U*_eq_(C) or 1.5*U*_eq_(methyl C).

### Crystal data

**Table:**

C_17_H_18_O_5_	*F*_000_ = 640
*M**_r_* = 302.31	*D*_x_ = 1.327 Mg m^−^^3^
Monoclinic, *P*2_1_/*c*	Mo *K*α radiation λ = 0.71073 Å
Hall symbol: -P 2ybc	Cell parameters from 1217 reflections
*a* = 14.431 (3) Å	θ = 1.6–24.7º
*b* = 14.073 (3) Å	µ = 0.10 mm^−^^1^
*c* = 7.4610 (15) Å	*T* = 293 (2) K
β = 92.86 (3)º	Block, light-yellow
*V* = 1513.3 (5) Å^3^	0.30 × 0.30 × 0.30 mm
*Z* = 4	

### Data collection

**Table:**

Enraf–Nonius CAD-4 diffractometer	2667 independent reflections
Radiation source: fine-focus sealed tube	1543 reflections with *I* > 2σ(*I*)
Monochromator: graphite	*R*_int_ = 0.059
*T* = 293(2) K	θ_max_ = 25.0º
ω/2θ scans	θ_min_ = 1.4º
Absorption correction: ψ scan(North *et al.*, 1968)	*h* = −17→17
*T*_min_ = 0.971, *T*_max_ = 0.971	*k* = −16→0
2893 measured reflections	*l* = 0→8

### Refinement

**Table:**

Refinement on *F*^2^	Secondary atom site location: difference Fourier map
Least-squares matrix: full	Hydrogen site location: difmap and geom
*R*[*F*^2^ > 2σ(*F*^2^)] = 0.071	H atoms treated by a mixture of independent and constrained refinement
*wR*(*F*^2^) = 0.215	*w* = 1/[σ^2^(*F*_o_^2^) + (0.1231*P*)^2^] where *P* = (*F*_o_^2^ + 2*F*_c_^2^)/3
*S* = 1.03	(Δ/σ)_max_ < 0.001
2667 reflections	Δρ_max_ = 0.28 e Å^−^^3^
206 parameters	Δρ_min_ = −0.35 e Å^−^^3^
Primary atom site location: structure-invariant direct methods	Extinction correction: none

### Special details

**Table:**

Geometry. All e.s.d.'s (except the e.s.d. in the dihedral angle between two l.s. planes) are estimated using the full covariance matrix. The cell e.s.d.'s are taken into account individually in the estimation of e.s.d.'s in distances, angles and torsion angles; correlations between e.s.d.'s in cell parameters are only used when they are defined by crystal symmetry. An approximate (isotropic) treatment of cell e.s.d.'s is used for estimating e.s.d.'s involving l.s. planes.
Refinement. Refinement of *F*^2^ against ALL reflections. The weighted *R*-factor *wR* and goodness of fit *S* are based on *F*^2^, conventional *R*-factors *R* are based on *F*, with *F* set to zero for negative *F*^2^. The threshold expression of *F*^2^ > σ(*F*^2^) is used only for calculating *R*-factors(gt) *etc*. and is not relevant to the choice of reflections for refinement. *R*-factors based on *F*^2^ are statistically about twice as large as those based on *F*, and *R*- factors based on ALL data will be even larger.

### Fractional atomic coordinates and isotropic or equivalent isotropic displacement parameters (Å^2^)

**Table:**

	*x*	*y*	*z*	*U*_iso_*/*U*_eq_	
C1	0.4333 (2)	0.7518 (2)	1.0256 (5)	0.0384 (8)	
C2	0.3890 (2)	0.6623 (2)	1.0334 (4)	0.0379 (8)	
C3	0.2979 (2)	0.6555 (2)	1.0813 (4)	0.0391 (8)	
C4	0.2496 (2)	0.7362 (2)	1.1277 (4)	0.0399 (9)	
C5	0.2918 (2)	0.8252 (2)	1.1218 (4)	0.0396 (8)	
H5	0.2595	0.8796	1.1519	0.048*	
C6	0.3819 (2)	0.8313 (2)	1.0709 (5)	0.0431 (9)	
H6	0.4097	0.8909	1.0666	0.052*	
C7	0.6747 (2)	0.8578 (2)	0.9220 (5)	0.0401 (9)	
C8	0.7407 (3)	0.8069 (3)	1.0250 (5)	0.0504 (10)	
H8	0.7216	0.7680	1.1170	0.060*	
C9	0.8331 (3)	0.8129 (3)	0.9935 (5)	0.0562 (11)	
H9	0.8758	0.7771	1.0621	0.067*	
C10	0.8632 (2)	0.8713 (3)	0.8614 (6)	0.0486 (10)	
C11	0.7995 (3)	0.9231 (3)	0.7578 (5)	0.0468 (9)	
H11	0.8191	0.9628	0.6674	0.056*	
C12	0.7062 (3)	0.9155 (2)	0.7898 (5)	0.0442 (9)	
H12	0.6635	0.9506	0.7196	0.053*	
C13	0.5738 (2)	0.8556 (3)	0.9633 (5)	0.0488 (10)	
H13A	0.5397	0.8940	0.8743	0.059*	
H13B	0.5672	0.8858	1.0789	0.059*	
C14	0.5283 (2)	0.7592 (2)	0.9682 (4)	0.0398 (9)	
C15	0.2723 (3)	0.5045 (3)	1.2103 (6)	0.0663 (12)	
H15A	0.3383	0.4967	1.2260	0.100*	
H15B	0.2441	0.4444	1.1807	0.100*	
H15C	0.2483	0.5281	1.3194	0.100*	
C16	0.1069 (3)	0.8046 (3)	1.2136 (6)	0.0631 (12)	
H16A	0.1365	0.8398	1.3107	0.095*	
H16B	0.0464	0.7849	1.2464	0.095*	
H16C	0.1012	0.8440	1.1086	0.095*	
C17	0.9921 (4)	0.9345 (4)	0.7095 (9)	0.101 (2)	
H17A	0.9633	0.9206	0.5938	0.152*	
H17B	1.0580	0.9260	0.7057	0.152*	
H17C	0.9790	0.9991	0.7412	0.152*	
O1	0.57164 (18)	0.68781 (18)	0.9225 (4)	0.0543 (8)	
O2	0.4326 (2)	0.58075 (17)	0.9921 (4)	0.0499 (7)	
O3	0.25205 (18)	0.56972 (17)	1.0703 (3)	0.0511 (7)	
O4	0.16164 (17)	0.72248 (18)	1.1765 (4)	0.0517 (7)	
O5	0.95673 (19)	0.8722 (2)	0.8396 (5)	0.0760 (10)	
H18	0.489 (3)	0.597 (3)	0.960 (6)	0.063 (14)*	

### Atomic displacement parameters (Å^2^)

**Table:**

	*U*^11^	*U*^22^	*U*^33^	*U*^12^	*U*^13^	*U*^23^
C1	0.0403 (19)	0.0405 (19)	0.0339 (19)	0.0015 (16)	−0.0042 (15)	−0.0021 (15)
C2	0.050 (2)	0.0317 (18)	0.0310 (18)	0.0016 (15)	−0.0098 (15)	−0.0011 (14)
C3	0.046 (2)	0.0368 (19)	0.0338 (19)	−0.0040 (16)	−0.0085 (15)	0.0029 (15)
C4	0.040 (2)	0.045 (2)	0.0335 (19)	−0.0004 (16)	−0.0080 (15)	0.0018 (16)
C5	0.043 (2)	0.0360 (19)	0.040 (2)	0.0025 (15)	−0.0019 (16)	−0.0027 (15)
C6	0.053 (2)	0.0343 (19)	0.042 (2)	−0.0029 (16)	−0.0027 (17)	−0.0033 (16)
C7	0.045 (2)	0.0362 (18)	0.039 (2)	−0.0011 (16)	0.0019 (16)	−0.0038 (16)
C8	0.054 (2)	0.052 (2)	0.044 (2)	−0.0035 (19)	0.0015 (18)	0.0173 (18)
C9	0.052 (2)	0.058 (2)	0.057 (3)	0.0054 (19)	−0.0058 (19)	0.019 (2)
C10	0.041 (2)	0.040 (2)	0.066 (3)	0.0021 (17)	0.0118 (19)	0.0042 (19)
C11	0.055 (2)	0.040 (2)	0.046 (2)	0.0043 (18)	0.0107 (18)	0.0073 (17)
C12	0.053 (2)	0.0353 (19)	0.044 (2)	0.0051 (17)	−0.0011 (17)	0.0024 (16)
C13	0.046 (2)	0.045 (2)	0.056 (2)	−0.0046 (18)	0.0009 (18)	−0.0055 (19)
C14	0.046 (2)	0.042 (2)	0.0307 (19)	0.0023 (16)	−0.0067 (15)	−0.0015 (16)
C15	0.087 (3)	0.048 (2)	0.063 (3)	−0.012 (2)	−0.004 (2)	0.011 (2)
C16	0.045 (2)	0.063 (3)	0.081 (3)	0.006 (2)	0.001 (2)	−0.001 (2)
C17	0.065 (3)	0.081 (4)	0.162 (6)	0.008 (3)	0.047 (4)	0.047 (4)
O1	0.0498 (16)	0.0450 (15)	0.0682 (19)	−0.0002 (12)	0.0049 (13)	−0.0119 (13)
O2	0.0490 (17)	0.0357 (14)	0.0643 (18)	0.0020 (12)	−0.0038 (14)	−0.0076 (12)
O3	0.0600 (17)	0.0372 (14)	0.0544 (17)	−0.0103 (12)	−0.0131 (12)	0.0028 (12)
O4	0.0407 (15)	0.0497 (15)	0.0645 (18)	−0.0039 (12)	0.0017 (12)	−0.0010 (13)
O5	0.0485 (18)	0.075 (2)	0.106 (3)	0.0105 (16)	0.0207 (16)	0.0276 (19)

### Geometric parameters (Å, °)

**Table:**

C1—C6	1.393 (5)	C11—C12	1.383 (5)
C1—C2	1.415 (5)	C11—H11	0.9300
C1—C14	1.461 (5)	C12—H12	0.9300
C2—O2	1.351 (4)	C13—C14	1.509 (5)
C2—C3	1.383 (5)	C13—H13A	0.9700
C3—O3	1.378 (4)	C13—H13B	0.9700
C3—C4	1.386 (5)	C14—O1	1.240 (4)
C4—O4	1.352 (4)	C15—O3	1.410 (5)
C4—C5	1.394 (5)	C15—H15A	0.9600
C5—C6	1.376 (5)	C15—H15B	0.9600
C5—H5	0.9300	C15—H15C	0.9600
C6—H6	0.9300	C16—O4	1.434 (5)
C7—C12	1.372 (5)	C16—H16A	0.9600
C7—C8	1.393 (5)	C16—H16B	0.9600
C7—C13	1.505 (5)	C16—H16C	0.9600
C8—C9	1.368 (5)	C17—O5	1.422 (5)
C8—H8	0.9300	C17—H17A	0.9600
C9—C10	1.370 (5)	C17—H17B	0.9600
C9—H9	0.9300	C17—H17C	0.9600
C10—O5	1.368 (4)	O2—H18	0.89 (4)
C10—C11	1.380 (5)		
			
C6—C1—C2	117.3 (3)	C7—C12—H12	119.0
C6—C1—C14	122.2 (3)	C11—C12—H12	119.0
C2—C1—C14	120.5 (3)	C7—C13—C14	116.8 (3)
O2—C2—C3	117.4 (3)	C7—C13—H13A	108.1
O2—C2—C1	122.1 (3)	C14—C13—H13A	108.1
C3—C2—C1	120.5 (3)	C7—C13—H13B	108.1
O3—C3—C2	120.3 (3)	C14—C13—H13B	108.1
O3—C3—C4	119.1 (3)	H13A—C13—H13B	107.3
C2—C3—C4	120.4 (3)	O1—C14—C1	121.1 (3)
O4—C4—C3	116.2 (3)	O1—C14—C13	119.8 (3)
O4—C4—C5	123.7 (3)	C1—C14—C13	119.1 (3)
C3—C4—C5	120.1 (3)	O3—C15—H15A	109.5
C6—C5—C4	119.0 (3)	O3—C15—H15B	109.5
C6—C5—H5	120.5	H15A—C15—H15B	109.5
C4—C5—H5	120.5	O3—C15—H15C	109.5
C5—C6—C1	122.6 (3)	H15A—C15—H15C	109.5
C5—C6—H6	118.7	H15B—C15—H15C	109.5
C1—C6—H6	118.7	O4—C16—H16A	109.5
C12—C7—C8	117.4 (3)	O4—C16—H16B	109.5
C12—C7—C13	121.2 (3)	H16A—C16—H16B	109.5
C8—C7—C13	121.3 (3)	O4—C16—H16C	109.5
C9—C8—C7	121.2 (3)	H16A—C16—H16C	109.5
C9—C8—H8	119.4	H16B—C16—H16C	109.5
C7—C8—H8	119.4	O5—C17—H17A	109.5
C8—C9—C10	120.5 (4)	O5—C17—H17B	109.5
C8—C9—H9	119.7	H17A—C17—H17B	109.5
C10—C9—H9	119.7	O5—C17—H17C	109.5
O5—C10—C9	116.2 (3)	H17A—C17—H17C	109.5
O5—C10—C11	124.2 (3)	H17B—C17—H17C	109.5
C9—C10—C11	119.6 (3)	C2—O2—H18	107 (3)
C10—C11—C12	119.3 (3)	C3—O3—C15	116.4 (3)
C10—C11—H11	120.3	C4—O4—C16	118.1 (3)
C12—C11—H11	120.3	C10—O5—C17	118.5 (3)
C7—C12—C11	122.0 (3)		
			
C6—C1—C2—O2	180.0 (3)	C8—C9—C10—C11	−1.1 (6)
C14—C1—C2—O2	1.3 (5)	O5—C10—C11—C12	178.9 (4)
C6—C1—C2—C3	1.1 (5)	C9—C10—C11—C12	0.3 (6)
C14—C1—C2—C3	−177.6 (3)	C8—C7—C12—C11	0.4 (5)
O2—C2—C3—O3	−5.9 (5)	C13—C7—C12—C11	175.7 (3)
C1—C2—C3—O3	173.0 (3)	C10—C11—C12—C7	0.0 (6)
O2—C2—C3—C4	179.1 (3)	C12—C7—C13—C14	128.6 (4)
C1—C2—C3—C4	−2.0 (5)	C8—C7—C13—C14	−56.2 (5)
O3—C3—C4—O4	6.4 (4)	C6—C1—C14—O1	−177.5 (3)
C2—C3—C4—O4	−178.6 (3)	C2—C1—C14—O1	1.2 (5)
O3—C3—C4—C5	−173.4 (3)	C6—C1—C14—C13	2.0 (5)
C2—C3—C4—C5	1.7 (5)	C2—C1—C14—C13	−179.3 (3)
O4—C4—C5—C6	179.7 (3)	C7—C13—C14—O1	−6.7 (5)
C3—C4—C5—C6	−0.5 (5)	C7—C13—C14—C1	173.8 (3)
C4—C5—C6—C1	−0.3 (5)	C2—C3—O3—C15	78.6 (4)
C2—C1—C6—C5	0.0 (5)	C4—C3—O3—C15	−106.3 (4)
C14—C1—C6—C5	178.7 (3)	C3—C4—O4—C16	−175.8 (3)
C12—C7—C8—C9	−1.1 (6)	C5—C4—O4—C16	4.0 (5)
C13—C7—C8—C9	−176.5 (4)	C9—C10—O5—C17	−177.4 (4)
C7—C8—C9—C10	1.5 (6)	C11—C10—O5—C17	3.9 (6)
C8—C9—C10—O5	−179.8 (4)		

### Hydrogen-bond geometry (Å, °)

**Table:**

*D*—H···*A*	*D*—H	H···*A*	*D*···*A*	*D*—H···*A*
O2—H18···O1	0.89 (4)	1.78 (4)	2.583 (4)	148 (4)
C15—H15A···O2	0.96	2.56	3.086 (5)	115
C11—H11···O3^i^	0.93	2.51	3.259 (4)	138
C17—H17B···O4^ii^	0.96	2.59	3.315 (6)	133

Symmetry codes: (i) −*x*+1, *y*+1/2, −*z*+3/2; (ii) *x*+1, −*y*+3/2, *z*−1/2.
